# Short-term residual effects of smoked cannabis on simulated driving performance

**DOI:** 10.1007/s00213-025-06880-1

**Published:** 2025-09-06

**Authors:** Kyle F. Mastropietro, Jake A. Rattigan, Anya Umlauf, David J. Grelotti, Marilyn A. Huestis, Raymond T. Suhandynata, Igor Grant, Robert L. Fitzgerald, Thomas D. Marcotte

**Affiliations:** 1 San Diego State University/University of California San Diego Joint Doctoral Program in Clinical Psychology, San Diego, CA USA; 2 Center for Medicinal Cannabis Research, Department of Psychiatry, University of California San Diego, San Diego, CA USA; 3 Institute for Emerging Health Professions, Thomas Jefferson University, Philadelphia, PA USA; 4 Center for Medicinal Cannabis Research, Department of Pathology, University of California San Diego, San Diego, CA USA

**Keywords:** Cannabis, Marijuana, Driving, Residual effects

## Abstract

**Rationale:**

Between periods of use, chronic cannabis consumers may display residual effects on selective cognitive functions, particularly memory and attention. Whether there are comparable deficits in real-world behaviors, such as driving, has not been thoroughly examined.

**Objectives:**

The current study explored the association between driving simulator performance, cannabis use history, and demographic factors after *≥* 48 h of abstinence. Study I examined simulator performance across a broad range of use within 191 healthy cannabis users. Study II compared performance between participants with the highest cannabis use intensity and a non-cannabis-using comparison group.

**Methods:**

In Study I, 191 healthy cannabis users completed a 25-minute simulated drive, following *≥* 48 h of abstinence. In Study II, a pilot study comprising a subset of 18 frequent cannabis users was compared to 12 non-using controls who completed identical driving measures in a separate study. In both studies, the main outcome was the Composite Drive Score (CDS), a global measure of driving performance comprising key driving-related variables, including standard deviation of lateral position.

**Results:**

In Study I, there was no relationship between CDS, its subtests, measures of cannabis use history, or demographic variables (all *p*s > 0.10). In Study II, frequent cannabis users and the non-using comparison group did not differ on CDS or performance on its subtests (all *p*s > 0.40).

**Conclusions:**

The current study did not find evidence of a residual effect of cannabis on simulated driving performance during a short period of cannabis abstinence. Future studies would benefit from inclusion of larger non-cannabis-using comparison groups.

**Supplementary Information:**

The online version contains supplementary material available at 10.1007/s00213-025-06880-1.

## Introduction

With cannabis use increasing in the United States and internationally (Substance Abuse and Mental Health Services Administration 2020; United Nations Office on Drugs and Crime 2021), it is important to examine the full range of effects of cannabinoids, especially Δ9-tetrahydrocannabinol (THC), on real-world functioning. Previous research established that acute THC consumption may result in decrements in aspects of cognition including memory and attention (Broyd et al. 2016; Gonzalez et al. 2017). Furthermore, acute smoked cannabis use can impair driving simulator performance, resulting in increased swerving (standard deviation of lateral position; SLDP) and decreased ability to adjust speed in relation to a lead vehicle (i.e., car following; Hartman et al. 2015; Ramaekers 2018; Marcotte et al. 2022). Various studies also probed the nature of possible residual deleterious effects that persist during periods of cannabis abstinence. Meta-analyses found residual effects on learning and recall after unknown periods of abstinence (Grant et al. 2003; Schreiner and Dunn 2012), although effect sizes were generally small. Some found that decrements in global cognition alleviated within 25 days of abstinence (Schreiner and Dunn 2012), while others determined that episodic memory remains negatively impacted beyond 28 days of abstinence (Crane et al. 2013). Similarly, reductions in memory and attention were seen following several days and weeks of abstinence in frequent, chronic users (Pope Jr. et al. 2001; Broyd et al. 2016). Furthermore, one inpatient study also found that critical tracking and divided attention improved from baseline but remained worse when compared to controls following 28 days of abstinence, although the user and control groups were not matched on key demographics (Bosker et al. 2013). Current knowledge gaps in the field include whether potential short-term residual effects immediately following cannabis abstinence negatively impact real-world activities such as driving.

Only a limited number of studies explored whether short-term residual effects are seen with driving performance. Since most controlled studies examine the administration of cannabis to participants, participants typically consist of current cannabis users, and exclude non-users as a potential comparison group, with few exceptions. One study analyzed simulated driving performance in 28 heavy, chronic cannabis users with at least 12 h of self-reported abstinence, and 16 healthy non-using controls (Dahlgren et al. 2020). Using a one-tailed t-test, cannabis users evidenced significantly worse performance across numerous outcomes. When stratifying users based upon age of use onset, group differences were driven by those who started using prior to age 16. Upon controlling for impulsivity, most of the driving differences between early-onset users and controls were attenuated, suggesting some of the differences may be attributable to pre-morbid influences. However, this study had several limitations: exact length of abstinence periods was unknown, the driving task was brief, and objective confirmation of cannabis abstinence was not obtained. In another simulator study with non-using controls, SDLP and speed relative to the posted speed limit were examined in a sample of 31 heavy, daily cannabis users and 24 occasional users following at least 8–12 h of self-reported abstinence, as compared with 30 non-using controls (Brooks-Russell et al. 2021). Although their analysis did not intend to investigate differences in baseline SDLP, the authors found that both cannabis using groups displayed lower (better) baseline SDLP than the control group.

Previous studies also suggested that short-term residual effects are most likely to manifest for frequent cannabis users (Grant et al. 2003). One meta-analysis found that short-term residual effects on memory were most pronounced in “heavy” cannabis users (i.e., meeting criteria for cannabis dependence, using > 20 times per month, or using > 30 joints per month) with abstinence periods shorter than 10 days (Schoeler et al. 2016). These results were supported by individual studies finding, for example, that long-term users (median 2 joints/day, 27.9 days/month) evidenced worse cognitive performance than short-term users and controls following a median of 17 h of abstinence (Solowij et al. 2002), and current users (median 19 years using cannabis *≥* 7 times per week) evidenced worse cognitive performance than former users and controls up to seven days after initiation of abstinence (Pope Jr et al. 2001). Still, others were able to explore residual effects in users with extremely high use intensity (mean of approximately 93 joints per week), finding that these users displayed worse cognitive performance even after 28 days of abstinence compared to participants with lighter use intensity (Bolla et al. 2002).

The current study seeks to build upon previous findings and examine whether short-term residual cannabis effects manifest as worse driving performance in the absence of acute intoxication. The paper reports on two studies: Study I probed the existence of a dose-response relationship with respect to short-term residual adverse effects on driving simulator performance in a sample of 191 current cannabis users who were part of a large randomized, placebo-controlled clinical trial on acute cannabis exposure (Marcotte et al. 2022). Users exhibited a range of use histories and abstinence periods. The aim of Study I was to determine whether performance on the Composite Drive Score (CDS), a measure encapsulating key variables of interest in assessing driving simulator performance, is related to cannabis use intensity within the 6 months prior to enrollment, days of abstinence, age of cannabis use onset, or baseline blood THC concentrations, with the assumption that residual effects would likely vary according to intensity of use. In Study II, potential short-term residual effects of cannabis on driving simulator performance for the most frequent cannabis users in Study I (*n* = 18) was compared with driving simulator performance in a healthy non-cannabis-using comparison group (*n* = 12) recruited for a different study. The most frequent users from Study I, who consumed cannabis on at least 28 of the previous 30 days *and* *≥* 1 gram of cannabis per smoking day, were selected for Study II analyses. Study II specifically examined whether there are any indications of residual effects on driving in the most intense cannabis users versus a healthy non-using comparison group.

## Materials and methods

### Participants

Recruitment of cannabis-using participants occurred from February of 2017 to June of 2019 in the San Diego, CA area using flyers disseminated in local dispensaries and venues, community outreach, and clinicaltrials.gov. Key inclusion criteria were being 21–55 years of age, cannabis use *≥* 4 times in the last month, a valid driver’s license, driving at least 1,000 miles in the past year, no self-reported history of serious mental or physical illness, and willingness to abstain from cannabis for 48 h prior to the training and experimental days.

In Study II, a convenience sample of healthy non-cannabis-using adults (*n* = 12) were recruited from a study of the effects of acute alcohol administration (*N* = 22) whose key inclusion criteria were being aged 21–55, holding an active driver license and driving *≥* 1,000 miles within the previous year, and occasional alcohol use (*≥* 3 times within the past month). For inclusion in Study II, the non-using comparison group was also required to self-report no more than a mild alcohol use history (defined as drinking < 15 of the previous 30 days) and have not used cannabis within the past year given the literature showing any residual effects typically resolve within 30 days since the last use (Schreiner and Dunn 2012; Crane et al. 2013). Only four participants reported previous cannabis use, the most recent being 365 days prior to enrollment. Attempting to harmonize the various definitions of high cannabis use intensity from the literature, only Study I participants consuming cannabis for at least 28 of the previous 30 days and consuming an average of at least 1 gram of cannabis per smoking day i.e., those with the highest use intensity of all 191 participants, were included in this analysis (*n* = 18).

Exclusion criteria for all participants were history of traumatic brain injury or other neurological conditions, significant cardiovascular, hepatic, or renal disease, uncontrolled hypertension, and/or chronic pulmonary disease as assessed by a study physician, positive pregnancy test, positive urine toxicology screen for cocaine, amphetamines, opiates, and phencyclidine, past year substance use disorder, history of schizophrenia, bipolar disorder, and/or current suicidal ideation. For the cannabis-using group, those with an oral fluid (OF) THC > 5ng/mL on the testing day were excluded. Blood and oral fluid cannabinoid concentrations were quantified using isotope dilution liquid chromatography with tandem mass spectrometry (LC-MS/MS) as previously described (Sobolesky et al. 2019; Hubbard et al. 2020). All participants provided written informed consent.

### Study design

The current study was conducted as part of a randomized clinical trial on the effects of acute cannabis exposure on driving (Marcotte et al. 2022). For the cannabis-using participants in both phases, analyses are based upon simulator performance prior to cannabis or placebo administration. Cannabis-using participants were instructed to refrain from consuming cannabis 48 h prior to their training and experimental sessions, and abstinence was confirmed via analysis of OF samples using the Drager DrugTest 5000 as well as LC-MS/MS. The training session occurred following an in-person screening visit. During the training session, participants completed a practice driving simulation including all tasks they would encounter during the experimental session. Following training, participants completed a 25-minute simulated drive analogous to the simulated drive completed during experimental sessions. Both cannabis-using participants and controls completed identical training and experimental sessions on the same driving simulator.

### Substance use screening

During both the training and experimental session, all participants in both studies first completed a urine toxicology screen, breathalyzer, and OF sample to test for recent use of cannabis, alcohol, or other illicit substances. If a participant had OF THC levels *≥* 5ng/mL or breath alcohol concentration (BAC) > 0, indicating recent cannabis or alcohol use, their experimental session was cancelled.

### Driving simulation

Cannabis users, prior to smoking during the experimental session, and the non-using comparison group completed one 25-minute driving simulation presented on a STISIM M300WS-Console Driving Simulator System (Systems Technology, Inc; Hawthorne, CA) consisting of 3-screen, wide field-of-view monitors, steering wheel, and accelerator and brake pedals, and programmed using STISIM Drive v3.14 (Rosenthal 2017). The simulation included urban and rural driving segments, incorporating traffic challenges such as making left turns across oncoming traffic and crash avoidance. Variables commonly employed in substance-impaired driving literature e.g., SDLP and car following (Ramaekers et al. 2000; Hartman et al. 2015; Arkell et al. 2020) as well as variability in speed and number of correct responses on the Modified Surrogate Reference Task (mSuRT), a divided attention task adapted from the Surrogate Reference Task were also included (International Organization for Standardization 2019; Online Resource 1). The mSuRT task required participants to maintain their position and speed within their lane while touching circles of differing sizes that appeared on an iPad aside the driving simulator’s monitors. At another timepoint during the driving task, participants completed a car following task, with the primary outcome being coherence, or correlation, between the lead and participant car speed changes (Online Resource 1). These variables were aggregated into the CDS (similar to an overall z score), by combining multiple measures providing a stable global measure of driving performance which is not dependent on performance on any single component of the driving task (Wood 2002; Ortiz-Peregrina et al. 2020). The CDS therefore addresses concerns regarding the use of multiple dependent outcomes in studies of cannabis and driving (Alvarez et al. 2021). A higher CDS reflected poorer driving simulator performance.

### Data analysis

**Fig. 1:**
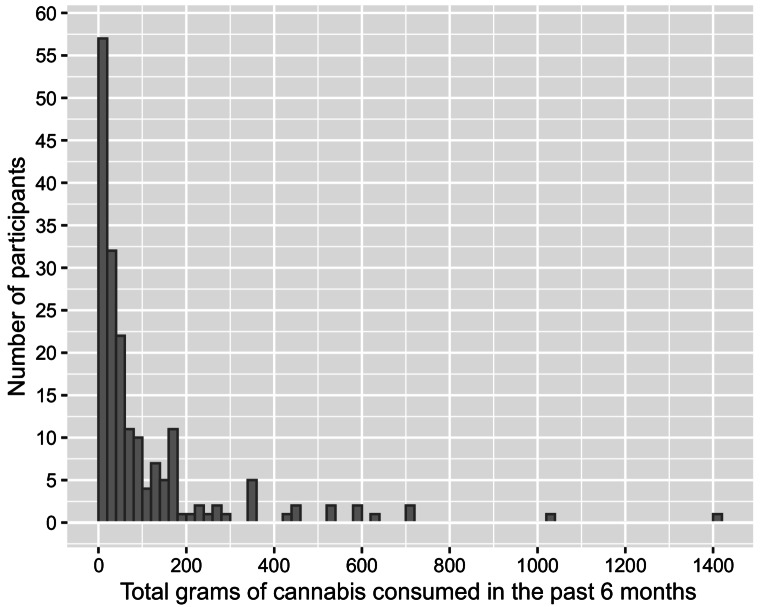
Distribution of estimated grams of cannabis consumed in the past 6 months for all cannabis-using participants (*N* = 191)

For Study I, cannabis use intensity (i.e., estimated total grams of cannabis consumption) was based upon self-reported frequency and quantity of use in the past 6 months during a timeline follow-back interview (Robinson et al. 2014; Fig. 1). First, the relationship between CDS and both estimated total grams of cannabis use and grams of cannabis use per day over the previous six months were analyzed. Participants were then split into tertiles based upon estimated grams of cannabis exposure over the last six months (lowest quartile, two middle-quartiles combined, and highest quartile) for an analysis of differences in CDS. Additional analyses of CDS differences included grouping cannabis-using participants based on the 15% of participants displaying the worst driving performance versus the upper 85%, age of cannabis use onset, sex, per se whole blood THC limits employed by law enforcement to detect on-road impairment (Asbridge 2014), and THC-COOH blood concentrations used in previous studies to differentiate heavy and occasional users (Fabritius et al. 2014). Subsequent analyses probed the relationship between overall CDS performance and performance on its subtests, cannabis use intensity variables spanning the previous 30 days and six months, and whole blood THC and metabolite concentrations. In Study II, frequent cannabis users were compared to the non-using comparison group on CDS and performance on its subtests, sex, race/ethnicity, age, miles driven within the past year, and years of education.

In Study I, bivariate Pearson correlation analyses were conducted to assess the relationships among the CDS, its subtests, cannabis use intensity over the previous 30 days and six months, age of use onset as a continuous variable, and blood cannabinoid concentrations. Spearman’s rho correlations were conducted for bivariate correlation analyses where outliers were present. One-way analyses of variance (ANOVAs) were conducted to analyze differences in driving performance variables using groupings based upon cannabis use intensity over the previous six months, age of use onset, demographic variables, and whole blood THC and metabolite concentrations. Analyses regarding days of cannabis abstinence included only 159 participants and analyses of age of cannabis use onset included only 146 participants due to these data being collected after some participants had already completed the study.

In Study II, one-way analyses of variance (ANOVAs) were conducted to analyze differences between frequent cannabis users and the non-using comparison group on CDS and performance on its subtests, age, number of miles driven within the last year, and years of education. Chi-square analyses were conducted to analyze group differences in demographic variables including sex and race. For the non-using comparison group, a bivariate Pearson correlation was conducted to analyze the relationship between days since last alcohol use and CDS to test for potential alcohol withdrawal effects (Jesse et al. 2017). All analyses were conducted using JMP Pro 16 statistical software. All figures were created using ggplot2 package (v3.5.1) in statistical software R (v4.2.1).

## Results

### Study I – Cannabis-using participants only

261 participants were screened for eligibility. During screening, 39 participants were excluded who did not meet eligibility criteria and 23 withdrew due to no longer being interested in participation. Seven participants were excluded the day of their experimental session for having OF THC > 5ng/mL and one participant withdrew on the experimental day, resulting in 191 participants being included in the analysis (Marcotte et al. 2022). Data for analyses were from the pre-smoking driving simulation completed during the experimental session. The final sample had a median age of 28 (21–54) years with 61.8% identifying as male and 38.2% as female, and 56.5% of participants identifying as racial/ethnic minorities (Table 1). Regarding driving history, the median miles driven in the past year was 8,730 miles [IQR 5,160, 13,280].

**Table 1:** Demographic breakdown of cannabis-using participants (*N* = 191)

Age^a^ (years)	31 (8.6)
Sex^b^	
Male	118 (61.8%)
Female	73 (38.2%)
Education^a^ (years)	15 (2)
Race/Ethnicity^b^	
Non-Hispanic White Hispanic	83 (43.3%)
Hispanic	56 (29.3%)
African American	18 (9.4%)
Asian	17 (8.9%)
Indigenous	8 (4.1%)
Multiracial	7 (3.6%)
Unknown	2 (1.0%)
Mile driven past year^c^	8,730 [IQR 5160, 13280]
Composite Drive Score^a^	0.01 (0.58)
Current cannabis use (< 4 times/week)^b^	98 (51.3%)
Days of cannabis use (last 30 days)^a^	16.7 (9.8) (~ 15%, 1 day/week)
Cannabis grams/day when using (last 30 days)^c^	0.5 [IQR 0.25, 1]
Total grams of cannabis used (last 30 days)^c^	6.78 [IQR 2, 20.19]
Cannabis grams/day when using (last 6 months)^c^	0.5 [IQR 0.25, 1]
Total grams of cannabis used (last 6 months)^c^	43.85 [IQR 17.02, 131]
Days of cannabis abstinence^a^	4.99 (3.94)
Age of cannabis use onset^a^	17.10 (4.57)

^a^Values reported as mean (standard deviation). ^b^Values reported as count (percentage). ^c^Values reported as median [interquartile range]

#### Use history and driving performance

Overall, 48.7% of participants used cannabis *≥* 4 times per week and 51.3% of the sample endorsed cannabis use < 4 times per week, with a mean of 16.7 days in the last 30 days (*SD* = 9.8; Table 1). Participants used a median of 0.5 g [IQR 0.25, 1] of cannabis per day in the last month. Approximately 15% endorsed consuming one day per week, with about 33% using 6–7 days per week. 72% of participants (*n* = 138) reported smoking as their primary route of cannabis administration and 28% of participants (*n* = 53) reported vaping as their primary route of administration. Participants were abstinent from cannabis for a mean(SD) of 4.99(3.94) days prior to their experimental session. 48% of participants endorsed abstinence periods *≤* 3 days and 27% of participants endorsed abstinence periods of 2 days. Overall, participant abstinence periods ranged from 2 to 21 days.

**Fig. 2:**
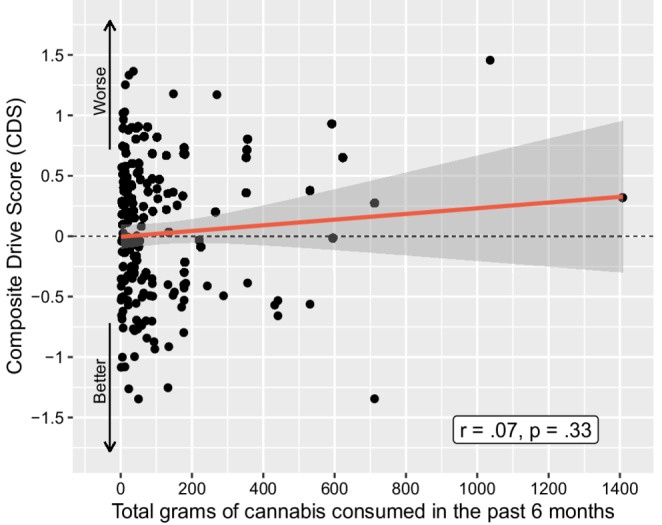
Correlation between Composite Drive Score (CDS) and estimated grams of cannabis consumed in the past 6 months for all cannabis-using participants (*N* = 191)

**Fig. 3:**
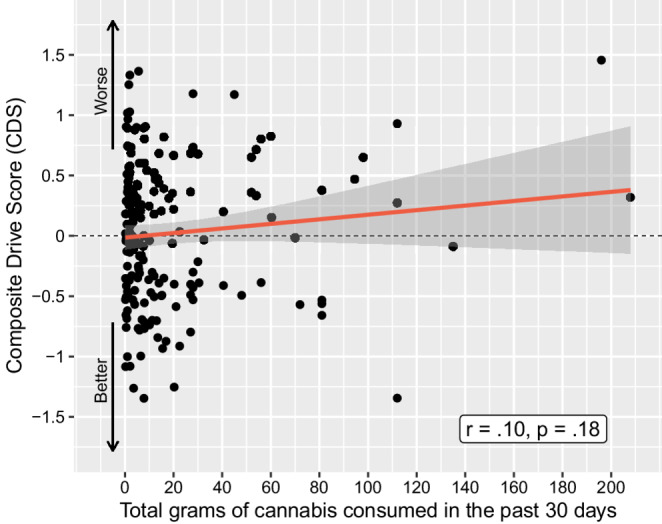
Correlation between Composite Drive Score (CDS) and estimated grams of cannabis consumed in the past 30 days for all cannabis-using participants (*N* = 191)

There was no relationship between cannabis use history and CDS performance pre-smoking, after an average of 5 days of abstinence. This included cannabis use intensity over the previous six months as a continuous variable (*r* =.07, *p* =.33; Fig. 2) and grams of cannabis use per day (*r* =.09, *p* =.22) over the past 6 months (Table 2). Looking broadly at use intensity within the entire sample, there was no difference in CDS when comparing participants who consumed cannabis *≥* 4 days per week within the last 30 days to those consuming cannabis < 4 days/week in the last 30 days, *p* =.96. Participants were then stratified into the three cannabis use intensity groups, with the lowest use group consuming a median of 6.5 g [IQR 3.56, 10.25], the middle use group consuming a median of 43.85 g [IQR 30.13, 66], and the highest use group consuming a median of 220.63 g [IQR 173.25, 434.25] over the previous 6 months. These use intensity groups did not differ on CDS, with mean(SD) CDS for the lowest = 0.056(0.595), middle 0.012(0.57), and highest 0.005(0.661) use intensity groups, respectively (*p* =.90). Examining use within the past 30 days, there were no relationships between CDS and number of days of use (*r* = −.04, *p* =.61), grams used per day (*r* =.05, *p* =.52), or total grams used (*r =*.10, *p* =.18, Fig. 3). Participants were further grouped by CDS for comparison of the 15% displaying the worst driving performance to the other 85%; there were no between-group differences regarding any cannabis use intensity or biological variables (all *p*s > 0.2).

**Table 2:** Correlations between key variables and composite drive score (*N* = 191)

	Composite Drive Score	
Use Intensity Variables	*r*	*p*
Grams/Day Over Previous 6 Months	0.09	0.22
Total Grams Over Previous 6 Months	0.07	0.33
Number of Days of Cannabis Use in Last 30 Days	− 0.04	0.61
Grams/Day Over Previous 30 Days	0.05	0.52
Total Grams Over Previous 30 Days	0.10	0.18
Days Since Last Use (*n* = 158)	0.03	0.66
Age of Onset (*n* = 146)	0.001	0.99
Blood Cannabinoid Concentrations	*r or Rho*	*p*
Whole Blood THC	− 0.05	0.50
11-OH-COOH^a, b^	− 0.08	0.31
THC-COOH^c^	− 0.04	0.59

^a^Spearman’s Rho is reported due to skewness of the data. ^b^11-hydroxy-THC. ^c^11-Nor-9-carboxy-THC

To explore the relationship between CDS and self-reported days of abstinence, data from 159 participants were analyzed (Table 2; 32 of the 191 participants completed the study prior to collection of these data). There was no relationship between days of abstinence and CDS (*r* =.03, *p* =.66). Participants endorsing higher use intensity reported shorter periods of abstinence (*r* = −.21, *p* =.01). 48% of participants endorsed cannabis abstinence periods *≤* 3 days and 27% of participants endorsed abstinence periods of 2 days. No differences emerged comparing participants with abstinence periods *≤* 3 days and > 3 days on the CDS and its subtests (all *p*s > 0.2) or mean driving speed (*p* =.64). Additionally, no differences emerged comparing participants with abstinence periods of 2 days and > 2 days on the CDS and its subtests (all *ps* > 0.47) or mean driving speed (*p* =.09).

#### Age of cannabis use onset

Given findings from previous literature suggesting age of cannabis use onset may predict short-term residual effects (Dahlgren et al. 2020), data from the 146 participants for whom age of use onset data existed were examined. The age of use onset as a continuous variable had no relationship with CDS (*r* =.001, *p* =.99; Table 2). Participants were further divided into two distinct groups based on their age of onset (before age 16 and at/after age 16). No significant difference in driving performance (*p* =.46) was seen between the earlier (mean(SD) CDS of 0.076(0.632)) and later (mean(SD) CDS of − 0.002(0.569)) onset users.

#### Subtests of the CDS

Considering performance on the subtests comprising the CDS, no relationships were found between SDLP (feet), coherence during the car-following task, standard deviation of speed (miles per hour), or mSuRT performance, and any cannabis use intensity variables over the previous 6 months, days of abstinence, or age of use onset (all *p*s > 0.10). No relationships were found among any of the subtests comprising the CDS and participant demographics (all *p*s > 0.10). Additionally, no relationships were found among any cannabis use intensity variables, days of abstinence, age of use onset, or demographic variables and mean driving speed (all *p*s > 0.15).

#### THC, metabolites, and driving performance

**Fig. 4:**
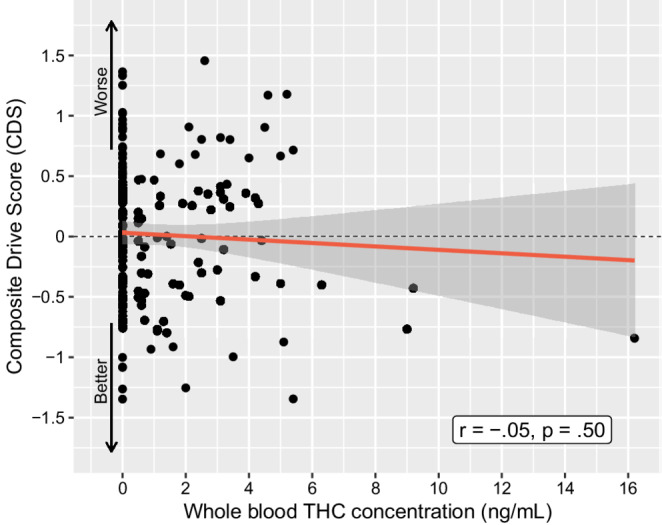
Correlation between Composite Drive Score (CDS) and whole blood THC concentration (ng/mL) for all cannabis-using participants (*N* = 191)

Regarding baseline biological indicators of past cannabis use, the mean(SD) whole blood concentrations (ng/mL) were: 1.2(2.0) ng/mL for THC, 0.5(2.0) ng/mL for 11-OH-COOH, and 12.0(20.3) ng/mL for THC-COOH. There was no relationship between the CDS and baseline cannabinoid concentrations: whole blood THC concentrations (*r = −*.05, *p* =.50, Fig. 4), 11-OH-COOH concentrations (Spearman’s rho = − 0.07, *p* =.31), or THC-COOH concentrations (*r* = −.04, *p* =.59; Table 2). No significant difference in CDS was found (*p* =.47) upon comparing participants with whole blood THC less than the lower limit of quantification (LLOQ), 0.5 ng/mL (*n* = 109; mean CDS = 0.042(0.058)) or greater than the LLOQ (*n* = 78; mean CDS = − 0.021(0.605)).

In response to the increasing legalization of recreational cannabis, some jurisdictions in the United States implemented per se limits, which are thresholds for the maximum amount of THC that may be present in one’s body while operating a vehicle (Asbridge 2014). One common per se cutoff for THC in blood as used by Washington, Colorado (using reasonable inference criteria), and Nevada is 5 ng/mL, although some literature suggested this cutoff was of limited relevance in predicting signs of cannabis intoxication (Hartman et al. 2016; Kosnett et al. 2023). Based on this cutoff, 10 of the 191 participants in this study (~ 5%) would be classified as under the influence despite reporting being abstinent from cannabis use for *≥* 48 h. No significant difference in CDS was found (*p* =.15) upon comparing participants with whole blood THC < 5 ng/mL (mean(SD) CDS = 0.03(0.571)) to those whose whole blood THC was *≥* 5 ng/mL (mean(SD) CDS = − 0.249(0.823)).

Previous studies also suggested that whole blood THC-COOH may differentiate heavy and occasional users and that whole blood concentrations *≥* 40 ng/mL indicate heavy cannabis use and whole blood concentrations *≤* 3 ng/mL suggest occasional consumption, although THC-COOH levels between these cutoffs were not reliably interpreted (Fabritius et al. 2014; Hoffman et al. 2021). Using these cutoffs, 18 participants (~ 9.5%) were classified as heavy users (mean concentration of 62.37 ng/mL (*SD* = 25.64)), 95 participants (50%) were classified as occasional users (mean concentration of 0.54 ng/mL (*SD* = 0.92)), and 77 participants (~ 40.5%) were classified as indeterminate users (mean concentration of 14.38 ng/mL (*SD* = 10.19)). These cutoffs were not significantly associated with poorer driving performance (*p* =.87), with a mean(SD) CDS of 0.08(0.75) for heavy users, 0.01(0.55) for indeterminate users, and 0.002(0.59) for occasional users.

### Study II – Cannabis users and non-using comparison group

Of a potential 22 healthy adult non-cannabis-using participants, 10 were excluded due to not meeting the alcohol use inclusion criteria for this phase or due to past-year cannabis use. At the time of enrollment, of the 12 remaining participants, only 4 had previously used cannabis in their lifetime and they were abstinent from cannabis for a median of 536 days [IQR 373, 10,866] at the time of enrollment. They had a median of 6.5 days [IQR 4, 8] of alcohol use and median of 13 drinks [IQR 8.5, 24] within the previous 30 days. They were abstinent from alcohol for a median of 3 days [IQR 1.25, 7] with a median of 833 days of lifetime alcohol use [IQR 294, 1,799]. The comparison group had a mean age of 33 years (*SD* = 13; median age = 28.5 [IQR 23, 47]; Table 3). Participants completed the same simulator training and simulated drive using the same driving simulator as the Study I participants, which was previously found to be sensitive to the intoxicating effects of acute cannabis use (Marcotte et al. 2022).

**Table 3:** Demographic breakdown of frequent cannabis users (*n* = 18; used 28 of the previous 30 days and *≥* 1 gram of cannabis per smoking day) and non-using comparison group (*n* = 12)

	Cannabis Users (*n* = 18)	Non-Using Comparison Group (*n* = 12)	*P* Value
Age^a^ (years)	32.7 (8.5)	33.3 (13.1)	0.88
Sex^b^			
Male	11 (61.1%)	8 (66.7%)	0.76
Female	7 (38.9%)	4 (33.3%)	
Education^a, *^ (years)	**13.4 (2.2)**	**15.8 (1.3)**	**< 0.01**
Race/Ethnicity^b^			
Non-Hispanic White	7 (38.8%)	8 (66.7%)	0.17
Hispanic	1 (5.6%)	0 (0%)	
African American	2 (11.1%)	0 (0%)	
Asian	1 (5.6%)	4 (33.3%)	
Indigenous	1 (5.6%)	0 (0%)	
Multiracial	6 (33.3%)	0 (0%)	
Unknown	0 (0%)	0 (0%)	
Miles driven past year^c^	10,000 [IQR 5,000, 24,200]	8,700 [IQR 4,050, 12,000]	0.09

^*^Bolded values reflect significant differences. ^a^Values reported as mean (standard deviation). ^b^Values reported as count (percentage). ^c^Values reported as median [interquartile range]

Of the 191 cannabis-using participants in Study I, 18 were identified who used cannabis at least 28 of the last 30 days and at least 1 gram of cannabis per smoking day. This group of frequent users consumed cannabis an average of 28.5 days within the last 30 days (*SD* = 0.92) and used an average of 2.30 g of cannabis, or about four 0.5 g joints, per smoking day (*SD* = 1.64). This is consistent with their self-report over the previous six months, where they consumed a median of 313 g of cannabis [IQR 178, 601] and used on average 2.32 g of cannabis, or about four 0.5 g joints, per smoking day (*SD* = 1.67). The median days of cannabis abstinence prior to testing was 3 days [IQR 2, 3]. At baseline, frequent cannabis users had a median whole blood THC concentration of 2.5 (0-9.2) ng/mL and a median blood THC-COOH concentration of 19.3 (1.8–83.9) ng/mL.

**Fig. 5:**
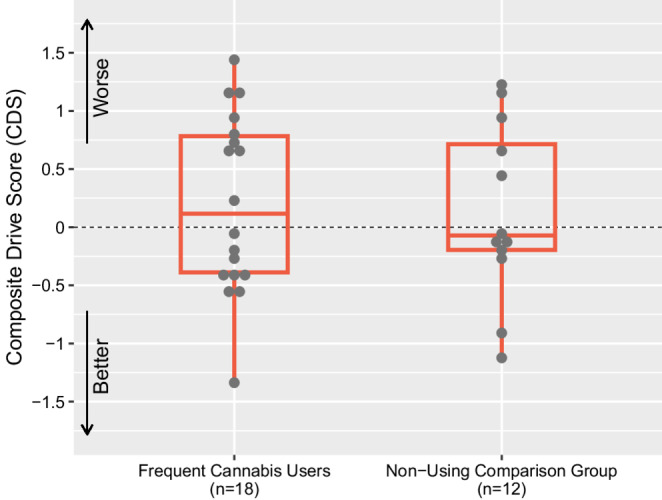
Comparison of Composite Drive Score (CDS) between frequent cannabis users (*n* = 18) and non-using comparison group (*n* = 12)

#### Driving performance and demographic variables

One-way ANOVAs revealed no significant differences between the comparison group and frequent cannabis users on the CDS: F(1,28) = 0.06, *p* =.81 (Fig. 5), nor on any of the subtests comprising the CDS or mean driving speed (miles per hour), all *p*s > 0.4 (Table 4). Participants in the comparison group, who were mild-moderate alcohol users, did not evidence alcohol withdrawal affecting driving performance, as days since last alcohol use (median = 3 [IQR 1.25, 7]) was not significantly related to CDS (*r* = −.55, *p* =.06). Comparison group participants and cannabis users did not differ on sex, race/ethnicity, age, nor on number of miles driven within the last year (all *p* >.09), although non-cannabis-using participants had significantly more years of education than cannabis users: F(1,28) = 11.27, *p* <.01, 15.83 (*SD* = 1.3) vs. 13.44 (*SD* = 2.2) years.

**Table 4:** Key driving outcomes in frequent cannabis users (*n* = 18) and Non-Using comparison group (*n* = 12)^a^

	Frequent Cannabis Users (*n* = 18)	Non-Using Comparison Group (*n* = 12)	*P* Value	Cohen’s d
Composite Drive Score (CDS)	0.2 (0.77)	0.13 (0.77)	0.81	− 0.09
Standard Deviation of Lateral Position (SDLP; feet)	1.19 (0.45)	1.17 (0.27)	0.85	− 0.07
Modified Surrogate Reference Task (mSuRT)	30.39 (1.5)	30.75 (0.97)	0.47	0.27
Car Following	0.69 (0.24)	0.62 (0.29)	0.51	− 0.25
Standard Deviation of Speed (miles per hour)	0.18 (0.81)	0.03 (0.82)	0.62	− 0.19
Mean Driving Speed (miles per hour)	62.43 (3.06)	62.59 (2.3)	0.88	0.06

^a^All values are reported as mean (standard deviation)

## Discussion

In regular cannabis users who abstained for *≥* 48 h and had oral fluid < 5ng/mL, we found no evidence of residual cannabis effects on simulated driving performance. This included no relationship between driving simulator performance and cannabis use intensity, days of abstinence, or cannabinoid concentrations, nor differences on these measures when comparing the most frequent cannabis users to a non-using comparison group. According to some of the earliest studies on this topic, short-term residual effects are best understood as lingering effects of cannabis on neuropsychological function present hours or days after one is no longer acutely intoxicated but during which the metabolites of cannabis may still be detected in blood or urine (Pope Jr. et al. 1995, 2001). Specifically, these effects may stem from either psychoactive metabolites of cannabis remaining in one’s central nervous system or from a persistent toxic effect of cannabis on the central nervous system that causes decrements in function. If residual effects are caused by THC or a psychoactive metabolite, a dose-dependent effect of variables such as higher use intensity, earlier age of onset of use, or shorter periods of abstinence would be expected (Bourque and Potvin 2021). However, in the current study, none of these factors were associated with decrements in simulated driving performance even when frequent cannabis users were compared to a non-using comparison group.

The current study adds to previous literature regarding the possible short-term residual effects of cannabis via the analysis of driving performance, cannabis use history, and demographic variables in a large cohort with a broad range of use patterns, and the comparison of these variables in a smaller subset of frequent cannabis users and a healthy non-using comparison group. While the current analyses focus on performance prior to administration of cannabis/placebo, examination of the current cohort following cannabis administration suggested that the deleterious effects of acute cannabis intoxication on driving performance largely dissipate about 4.5 h following consumption (Marcotte et al. 2022). The current study was able to probe whether short-term residual effects are evident in driving performance in the hours or days immediately after self-reported use. The present study also included variables not widely reported in the literature such as cannabis use history up to six months prior to enrollment, days of cannabis abstinence, age of cannabis use onset, and objective confirmation of ~ 24 h of cannabis abstinence as evidenced by an oral THC concentration of less than 5 ng/mL (Desrosiers et al. 2012).

Understanding whether there are residual cannabis effects remains critical, particularly to public safety. Several studies and meta-analyses found modest residual effects in specific cognitive tasks including verbal expression (Block and Ghoneim 1993), memory (Crane et al. 2013; Schoeler et al. 2016), attention (Fletcher et al. 1996; Pope Jr. et al. 2001; Broyd et al. 2016), and executive function (Pope Jr and Yurgelun-Todd 1996; Thames et al. 2014). Results from the current study suggest that residual cognitive deficits of cannabis, if present, may not translate directly to observable dysfunction in real-world, over-learned activities such as driving. It remains possible that residual cannabis effects may manifest during drives that are not laboratory-controlled and under infrequent real-world situations, such as braking in response to a sudden stimulus. However, analyzing granular and less common aspects of driving, such as reaction time associated with braking under emergency conditions may require repeated experimental trials and thus also sacrifice ecological validity.

### Limitations and future directions

The current study has limitations. Sample sizes included in Study II were relatively small and comparison of very frequent users to the non-using comparison group may have lacked adequate power to detect group differences. Using the best estimate we have for the standard deviation of CDS (0.58; Table 1), and assuming significance level α = 0.05 and 80% power, we estimate that a future study would need 394 participants per group to detect a mean difference in CDS of 0.12 (small Cohen’s d = 0.2), 64 participants per/group to detect a mean difference in CDS of 0.29 (medium Cohen’s d = 0.5), or 26 participants per group to detect a mean difference in CDS of 0.46 (large Cohen’s d = 0.8). The driving simulation employed addressed a subset of aspects of real-world driving performance, and thus may lack sensitivity to other driving-related abilities. The current study did not incorporate a measure of cannabis withdrawal symptoms and considering cannabis withdrawal symptoms may peak in the days following the initiation of cannabis abstinence (Haney 2005), it is unclear whether the results were confounded by potential participant experiences of withdrawal symptoms. However, no participants spontaneously reported such symptoms during the study and no relationships were found among cannabis use intensity, days of abstinence, and pre-smoking driving simulator performance. One study suggested impulsivity may be an important covariate in analyses of short-term residual effects of cannabis on driving and when including this in a model predicting driving performance, most differences between the group with a cannabis-use history and controls were attenuated (Dahlgren et al. 2020). The current study did not incorporate a measure of impulsivity. Furthermore, one systematic review of driving studies found that cannabis decreases reaction time among young adult users (Alvarez et al. 2021). The current study did not include a measure of reaction time. Additionally, it is possible that some of the cannabis using participants had abstinence periods shorter than 48 h since the Drager DrugTest 5000 may not detect THC in oral fluid beyond ~ 22 h since last use (Desrosiers et al. 2012). However, all cannabis users self-reported a minimum of 48 h of abstinence.

Some studies suggested that residual effects of cannabis may manifest on tests of verbal learning and memory 1–3 days after initiation of cannabis abstinence (Pope Jr. et al. 2001; Hanson et al. 2010). The current study was not designed to analyze residual effects after such brief timeframes due to including participants with a broad range of use patterns and requiring participants to refrain from cannabis use *≥* 48 h prior to their experimental session. It remains possible that residual effects may impact driving performance following shorter abstinence periods than those reported in the current study. However, it was suggested by one meta-analysis that residual effects of cannabis on neuropsychological performance tend to yield small, negative effect sizes that may not translate directly to disturbances in everyday functioning (Grant et al. 2003).

Since the current study was conducted in a well-controlled laboratory environment, it is unclear if the results are representative of on-road driving performance. Results from epidemiological studies analyzing the change in fatal and injurious crash rates from pre-to-post recreational cannabis legalization in states across the US are mixed, with some states reporting decreases in both and others reporting increases (Farmer et al. 2022). Notably, such studies are unable to draw conclusions about whether these trends are attributable to acute or residual effects of recreational cannabis on driving. Furthermore, due to constraints of utilizing a relatively short (25-minute) simulated drive, the traffic challenges presented during the trial may not have been complex or sensitive enough to capture observable decrements in driving performance resulting from residual effects. It is possible that if the simulated drive was longer and more complex as to selectively analyze sustained attention on driving performance, residual effects may have manifested. Lastly, some research has suggested that a blood THC-COOH concentration *≥* 40ng/mL reliably indicated heavy, chronic cannabis use (Fabritius et al. 2014). The mean(SD) blood concentration of THC-COOH at baseline for all 191 cannabis users was 12.0(20.3) ng/mL with only 17 participants testing *≥* 40 ng/mL, and thus according to these cutoffs, most cannabis-using participants evidenced lower use intensity than what some literature considers to be true frequent, chronic users for which short-term residual effects may be most likely to emerge.

## Conclusions

The current study showed no evidence of a dose-effect relationship between simulated driving performance following a brief abstinence period and critical variables such as cannabis use intensity within the last 6 months, age of use onset, length of abstinence periods, or baseline blood cannabinoid concentrations in a cohort of regular cannabis users. The current study also shows no evidence of short-term residual effects on simulated driving performance when comparing frequent cannabis users to a healthy non-using comparison group. Results of this study have implications for how future policy might weigh different pieces of evidence in the absence of objective confirmation of acute cannabis intoxication, such as one’s cannabis use history or residual blood THC, in everyday determinations of impaired driving. Future research should explore whether short-term residual effects exist for driving performance via the use of more complex driving tasks, incorporating larger non-using control groups, and better controlling for possible confounds such as cannabis withdrawal effects.

## Supplementary Information

Below is the link to the electronic supplementary material.

ESM1 (78.3 KB)

## Data Availability

Deidentified data is available for meta-analysis upon request to approved researchers with a signed data use agreement.
