# Supplementary material for: Short-term residual effects of smoked cannabis on simulated driving performance
Source: Psychopharmacology (Berl). 2025 Sep 6;243(4):793–804. doi: 10.1007/s00213-025-06880-1 (PMC12478537; doi:10.1007/s00213-025-06880-1)
Supplement: Supplementary file 1 — ESM1 (78.3 KB) [file 213_2025_6880_MOESM1_ESM.pdf]

Short-term Residual Effects of Smoked Cannabis on Simulated Driving Performance

Psychopharmacology

Kyle F. Mastropietro, MS[1,2]; Jake A. Rattigan, BA[1,2]; Anya Umlauf, MS[2];
David J. Grelotti, MD[2]; Marilyn A. Huestis, PhD[4]; Raymond T. Suhandynata, PhD[3];
Igor Grant, MD[2]; Robert L. Fitzgerald, PhD[3]; Thomas D. Marcotte, PhD[2`]

[1] San Diego State University/University of California San Diego Joint Doctoral Program in Clinical Psychology, San Diego, CA, United States of America
[2] Center for Medicinal Cannabis Research, Department of Psychiatry, University of California San Diego, San Diego, CA, United States of America
[3] Center for Medicinal Cannabis Research, Department of Pathology, University of California San Diego, San Diego, CA, United States of America
[4] Institute for Emerging Health Professions, Thomas Jefferson University, Philadelphia, PA, Unites States of America

Communicating Author:
Thomas D. Marcotte, PhD
tmarcotte@ucsd.edu

mSuRT

Divided attention was assessed by performance on the Modified Surrogate Reference Task, or mSuRT. The mSuRT required participants to maintain their position and speed on a straight road segment while responding to the task which was administered on an iPad to the side of the driving simulator's monitors. The mSuRT was initiated during the simulated drive by the sound of a phone ringing, and participants would then have to simultaneously maintain their speed on the road (65 mph) and correct position in the center of their lane while completing the task on an iPad off to the side of the simulator monitors. During the task itself, a pattern of random, hollow circles, with one of the circles being larger than the others was displayed on the iPad. The participant's task was to locate and touch the larger circle. The level of difficulty was varied by altering the ratio of the size of the distractor circles and target circles. To analyze mSuRT performance under controlled conditions, no other traffic or related road challenges were presented to participants during completion of the task. The mSuRT is ultimately a measure of performance under conditions of high cognitive load and controlled processing, where participants must divide their attention among three distinct stimuli and is analogous to real-life scenarios of divided attention during on-road driving (e.g., monitoring a GPS system).

Car following

Car following performance was assessed during a single segment of the simulated drive where participants were required to adjust their speed in response to a lead car that sped up and slowed down according to a sinusoidal

wave. The primary outcomes were coherence between the participant and lead car, the participant's ability to track changes in the lead car's speed, the reaction time for changes in speed in response to the lead car, and distance from the lead car.
